# Relationship between Life’s Essential 8 and metabolic syndrome among older Americans (NHANES, 2007–2010): navigating biological aging and inflammation

**DOI:** 10.3389/fmed.2024.1380464

**Published:** 2024-06-05

**Authors:** Ruoyu Gou, Si Xiong, Xudong Liang, Hao Wu, Shuitao Qin, Bing Li, Changjun Luo, Junan Chen

**Affiliations:** ^1^School of Public Health, Ningxia Medical University, Yinchuan, Ningxia, China; ^2^Department of Ultrasonography, Affiliated Liutie Central Hospital of Guangxi Medical University, Liuzhou, Guangxi, China; ^3^Department of Cardiology, Affiliated Liutie Central Hospital of Guangxi Medical University, Liuzhou, Guangxi, China; ^4^Department of Endocrinology, Affiliated Liutie Central Hospital of Guangxi Medical University, Liuzhou, Guangxi, China

**Keywords:** middle-aged and elderly people, metabolic syndrome, Life’s Essential 8, biological aging, inflammation

## Abstract

**Background:**

Metabolic syndrome (MetS) is a global health concern, and it is particularly harmful to middle-aged and elderly individuals. Life Element Eight (LE8), a measure to improve cardiovascular health, may offer benefits for MetS. Herein, we examined the relationship between LE8 and MetS among middle-aged and elderly individuals, and elucidated the role of biological aging and inflammation in this process.

**Methods:**

We obtained the LE8 scores of 2,901 Americans, along with their biological aging indicators (Biological age, Phenotypic age, Serum Klotho), and computed their inflammatory indicators SII, DII. Using logistic regression model, we assessed the association among inflammatory markers, Biological aging, LE8 and MetS. Additionally, we generated restricted cubic spline (RCS) plots to display trends in significant variables in logistic regression. Using parallel mediation analysis, we evaluated the possible mediating role of various factors in the risk relationship between LE8 and MetS.

**Results:**

Our examination revealed that higher LE8 scores were associated with a lower incidence of MetS in a fully adjusted model. The high LE8 subgroup had a 79.73% reduction in the risk of MetS compared to the low subgroup with an OR = 0.2027 (95% Cl 0.0871, 0.4714), with similar correlations between health factor scores and MetS risk. Biological aging mediated the associations between LE8, health behaviors and health factor scores and MetS risk.

**Conclusion:**

A rise in the LE8 score among middle-aged and elderly individuals is a protective factor for MetS, and this association may be partially mediated by biological aging, suggesting that LE8 may reduce the risk of MetS by ameliorating aging.

## 1 Introduction

Metabolic syndrome (MetS) refers to a range of metabolic disorders that involves augmented fasting blood glucose, hypertension, abdominal obesity, elevated triglycerides (TG) and diminished high-density lipoprotein cholesterol (HDL-C). MetS is a global public health problem, with a prevalence of about 25% worldwide and even higher in developed countries (1). According to the United States National Health and Nutrition Examination Survey (NHANES), over one third adults exhibit MetS, and the incidence rate among middle-aged and elderly people aged 40 and older is as high as 40% (2). MetS is particularly detrimental to middle-aged and elderly people, and some studies mainly composed of middle-aged and elderly people have suggested that MetS is associated with adverse cardiovascular risk and mortality (2, 3). At present, there are no MetS-targeting medications (3). Considering that MetS is closely correlated with cardiovascular disease (4), proper MetS management can potentially enhance cardio vascular health (CVH). More recently, the American Heart Association proposed novel CVH indicators, known collectively as Life’s Essential 8 (LE8) (5). LE8 involves diet, physical activity, nicotine exposure, sleep health, body mass index, lipids, blood glucose, and blood pressure. Relative to the Life’s Simple 7 score proposed in 2010, LE8 includes sleep quality and mental health assessment, and improved the scoring algorithm. Till date, there are no investigations that evaluated the relationship between the LE8 score and MetS, therefore, further research on the relationship between the two is necessary.

Aging is a strong modulator of vascular disease, which progresses to cardiovascular and cerebrovascular events, and it is one of the leading causes of patient deaths worldwide. Based on a recent investigation by Ma et al. (4), Americans with elevated LE8 scores lived longer than those with reduced LE8 scores. Thus, enhanced LE8 scores is linked to slowing down or blocking the aging process. Aging is not only associated with LE8, but also with MetS, which is characterized by insulin resistance, dyslipidemia, hypertension, and abdominal obesity (5), and which tends to accelerate aging, and we hypothesize that aging may partially mediate the association between MetS and LE8. In addition, there are now studies demonstrating a correlation between the pathogenesis of MetS and systemic inflammation. Tilg et al. (6) reported that MetS occurs due to excessive central obesity, which enhances adipose tissue CD8 (+) T cell aggregation, promotes macrophage recruitment and activation (7), and accelerates macrophage-driven metabolic disease inflammation (8). Given these evidences, blocking the inflammatory response is key to delaying MetS onset, and good cardiovascular health scores were shown to downregulate inflammatory markers (9). Therefore, we speculated that inflammation may be another factor that partially mediates the association between MetS and LE8. Based on these analyses, the present study conducted a cross-sectional study based on the 2007–2010 National Health and Nutrition Examination Survey (NHANES) to investigate the association between MetS and LE8 in middle-aged and older adults in the United States and the potential role of aging and chronic inflammation markers in mediating the association between MetS and LE8. potential mediating role in the association between MetS and LE8.

## 2 Methods

### 2.1 Study populations

NHANES is a continuing survey of the health and nutritional status of the United States population (10), and it is conducted by members of the Centers for Disease Control and Prevention (NCHS). Participant data are obtained via an intricate, classified, multistage probabilistic cluster sampling design, and involves 5,000 people each year. The collected information includes subject demographics, body measurements, laboratory test results, and dietary information (11). Prior to the survey, all subjects provided a written informed consent for survey participation and data usage in health-related statistical research.

Program details, data acquisition protocols, and available data are open to the public, and can be accessed at https://wwwn.cdc.gov/nchs/nhanes/. This investigation strictly followed the Strengthening the reporting of observational studies in epidemiology (STROBE) criteria for reported of observational studies in epidemiology (12). We examined 2 survey cycles spanning the years 2007–2010. Subjects were aged ≥ 40 with complete data, and among those excluded from analysis were participants with incomplete marital, family income-to-poverty ratio, education status, body mass index (BMI), smoking consumption status, alcohol consumption status, diabetes, phenotype age, SII, DII, LE8 or other information. Ultimately, we included 2,901 subjects in our analyses (Supplementary Figure 1).

### 2.2 Assessment of MetS

Adult Treatment Panel III (ATP III) of the National Cholesterol Education Program (NCEP) (3) states that MetS is the presence of ≥3 of the following characteristics: concentric obesity (with waist circumference > 35 inches or 88 cm among women and 40 inches or 102 cm among men), insulin resistance (fasting blood glucose > 100 mg/dL or lack of diabetes treatment), hypertension (SBP 130 mmHg or DBP 85 mmHg or requirement of hypertensive medication), elevated triglycerides (TG 150), and reduced high-density lipoprotein (HDL) cholesterol (HDL 40 among men and HDL 50 among women).

### 2.3 Assessment of LE8

The LE8 scoring system was based on the following questionnaires: 4 health behavior questions (diet, physical activity, nicotine exposure, and sleep duration) and 4 health parameters [BMI, non-HDL cholesterol, blood glucose (BG), and blood pressure (BP)]. The total score ranged between 0 and 100, and the overall LE8 score was calculated based on the average of 8 metrics. Detailed algorithms for calculating the LE8 scores for each of the metrics to NHANES data can be found in Supplementary Table 1. Following the American Heart Association definition, the LE8 scores were considered low (0–49 points), moderate (50–79 points), and high (80–100 points) (13). Dietary metrics were evaluated based on the 2015 Healthy Eating Index (HEI). Subjects were asked to recall two 24-h diets, and the data was integrated into the US Department of Agriculture (USDA) food pattern equivalence data to compute the HEI-2015 scores (14). Moreover, we utilized self-reports of the frequency and duration of intense or moderate physical activity over the past 30 days, and recorded the smoking status, sleep duration, diabetes history, medication history, BP, height, and weight during physical examination. BMI was defined as weight (kg) divided by height (m) squared. BP was recorded as the average reading of 3 consecutive measurements. Subjects were fasted for ≥8 h, prior to 5 mL blood sample collection, and the samples were dispatched to the central laboratory for complete blood count and biochemical (namely, blood lipids, BG, CRP and glycosylated hemoglobin) evaluation.

### 2.4 Assessment of inflammatory index (SII, DII)

Systemic inflammation was measured using both biochemical or hematological indicators in normal blood tests or as ratios from the measurements. SII was computed as follows: SII = P × N/L, whereby, P, N, and L were the preoperative peripheral platelet, neutrophil, and lymphocyte counts, respectively (15).

DII was first introduced in 2004, its first-generation protocols and verification were presented in 2009, and an enhanced version was offered in 2014 using comparative data from 11 national food and nutrition databases from 4 separate continents. We also measured relevant inflammatory markers, namely, CRP, IL-1β, IL-4, IL-6, IL-10, and TNF-α, to generate food parameter-specific DII scores, which, were, in turn, used to generate an overall DII score for individual subjects analyzed in this study (16).

The NHANES data comes with 28 available parameters that can be used to compute DII. These include energy, protein, carbohydrate, dietary fiber, total fatty acid, total saturated fatty acid, monounsaturated fatty acids (MUFA), polyunsaturated fatty acids (PUFA), cholesterol, β-carotene, niacin, folate, magnesium, iron, zinc, selenium, caffeine, alcohol, n3 polyunsaturated fatty acid, n6 polyunsaturated fatty acid, and vitamins A, B1, B2, B6, B12, C, D, and E. An augmented DII score represents an inflammation-triggering diets, and a reduced score represents an anti-inflammatory diet (17).

### 2.5 Evaluation of biological aging (biological age, phenotypic age, serum klotho)

We utilized the Klemera and Doubal method of calculating biological age using 8 biomarkers, namely, CRP, circulating creatinine, glycosylated hemoglobin, circulating albumin, circulating total cholesterol, circulating urea nitrogen, circulating alkaline phosphatase, and systolic BP (18).

The phenotypic age was computed using 9 age-related variables, namely, chronological age, albumin, creatinine, glucose, CRP, lymphocyte percent, average cell volume, red blood cell distribution width, alkaline phosphatase, and white blood cell count (19).

Circulating Klotho levels were assessed using ELISA (IBL International, Japan). All samples were twice assessed and the average of the two results were analyzed. Per plate, we also evaluated two quality control samples using reduced and elevated Klotho concentrations for accuracy assessment. The sample analyses were repeated if the assigned value was >2 standard deviations from the expected value. The assay sensitivity was 4.33 pg./mL, and both the intra- and inter-assay coefficient of variations were <5%. Please refer to https://wwwn.cdc.gov/Nchs/Nhanes/2007-2008/SSKL_E.htm for a detailed summary of the ELISA protocol (20).

### 2.6 Defining covariates

Covariates were defined as patient age, sex, ethnicity/race, marital status, family income to poverty ratio, education status, BMI, smoking consumption status, alcohol consumption status, and physical activity. Race was classified as white, black, Mexican, other. Education status was described as <11th grade, high school graduate, college graduate or above. Family income to poverty ratio was separated into 4 categories, based on a 1.3 to >5 scale as follows: 1.3, 1.3–3, 3–5, and >5. An augmented ratio represented a higher income status. Marital status was classified as follows: married, divorced, unmarried. Alcohol consumption frequency and consumer sex was recorded, and alcohol consumption was defined as: never, former, mild, moderate, and heavy (21). Smoking status can categorized as: currently smoking, formerly smoking, or never. Never smokers were those who smoked <than 100 cigarettes in their lives, ex-smokers were those who smoked > 100 cigarettes, but no longer smoking, and current smokers were defined as smoking > 100 cigarettes, with consistency or inconsistency (22). BMI was defined as follows (23): <25, 25–30, and >30 kg/m^2^. Physical activity (PA) can be divided into: People were asked about the types of exercise they did in the past month. These included walking, running, cycling, swimming, dancing, weightlifting, and other activities. The intensity of each activity was given as a metabolic equivalent (MET) and is in the original dataset. We multiplied the exercise frequency and its MET to get an activity score to evaluate the PA levels.

### 2.7 Statistical analysis

We employed the NHANES criteria for statistical analysis (oversampling, stratification, and clustering) to predict the appropriate number of U.S. adults to use in this study. In addition, we also evaluated the required statistical tests for weight adjustment. Categorical variables are presented in n (%), and inter-group differences were assessed via chi-squared test.

We also employed logistic regression models to assess relationships between inflammatory indices (SII, DII), biological aging (phenotypic age, circulating Klotho, biological age), and LE8 with MetS. To control for confounding factors, three weighted logistic regression models were used in this study: Crude model, No adjustment for any potential influence factors; Model 1, Adjusted for Sex, Age and Ethnic/race; Model 2, Adjusted for Sex, Age, Ethnic/race, Marital, Family income-to-poverty ratio Education levels and Alcohol consumption status. The acquired data are presented as weighted (OR [95% CI]). We also assessed sub-categories like patient gender, age, and ethnicity to further elucidate the aforementioned relationships within various populations. Herein, the LE8 (high, moderate and low CVH), health behavior (high, moderate and low CVH) and health factor (high, moderate and low CVH) scores were regarded as the reference group. We generated restricted cubic spline (RCS) plots to display patterns in variables of significance in logistic regression. Using the RCS plots, we determined the presence or absence of a nonlinear association between the mentioned exposure factors and MetS.

The possibility of the inflammatory index (SII, DII) and biological aging (phenotypic age, circulating Klotho, biological age) as a modulator of the LE8 and MetS association was further examined using a parallel mediation model employing a quasi-Bayesian Monte Carlo technique with 1,000 normal approximation-based simulations (R package “mediation”). Direct effect (DE) indicated the LE8-mediated regulation of MetS in the absence of mediators. Indirect effect (IE) indicated the LE8-mediated modulation of MetS using a mediator. Mediation was quantified as follows: IE divided by TE (total effect).

Various packages in R 4.2.2 (R Project for Statistical Computing), including, nhanesR (0.9.2.8), survey, compareGroups, dplyr, tidyverse, do, scatterplot3d, MASS, poLCA, finalfit, Hmisc, lattice, Formula, rms, and foreign were used to conduct the analyses. Significance was set at two-sided *p*-value < 0.05.

## 3 Results

### 3.1 Population demographics

Among the 2,901 subjects, 1,153 had a MetS diagnosis. Table 1 summarizes all participant demographics. In all, we observed marked differences in patient age, household income to poverty ratio, education status, BMI, smoking status, alcohol intake, physical activity, LE8 and LE8 components (health factors and health behaviors), various scores, including HEI, PA, Sleep, BMI, Non-HDL, Glucose, and BP, as well as phenotypic age, biological age, and DII among MetS and non-MetS subjects.

**Table 1:** Participant demographic characteristics (NHANES, 2007–2010 year cycle).

parameter	No. of participants (weighted %) a			
	Total	Non-MetS-ATP	MetS-ATP	*p*-value
	(*N* = 2, 901)	(*N* = 1, 748)	(*N* = 1, 153)	
**Sex**				
Female	1,397 (48.1558)	841 (51.4161)	556 (47.8690)	0.1811
Male	1,504 (51.8442)	907 (48.5839)	597 (52.1310)	
**Ethnicity/race**				
White people	1,672 (57.6353)	1,009 (80.7840)	663 (81.3175)	0.3618
Black people	447 (15.4085)	287 (7.6710)	160 (6.6853)	
Mexican	426 (14.6846)	234 (4.4629)	192 (5.4416)	
Other	356 (12.2716)	218 (7.0820)	138 (6.5556)	
**Marital**				
Married	1964 (67.7008)	1,199 (73.3899)	765 (73.8174)	0.5410
Separated	728 (25.0948)	407 (19.8342)	321 (20.5978)	
Never married	209 (7.2044)	142 (6.7759)	67 (5.5848)	
**Ratio of family income to poverty levels**				
<1.3	706 (24.3364)	383 (11.4583)	323 (16.7098)	<0.0010
1.3–3	868 (29.9207)	511 (22.9212)	357 (25.5613)	
3–5	620 (21.3719)	389 (25.6701)	231 (25.4612)	
≥5	707 (24.3709)	465 (39.9504)	242 (32.2676)	
**Education levels**				
Less than 11th grade	695 (23.9573)	387 (12.8552)	308 (16.5004)	0.0112
High school graduate	1,418 (48.8797)	889 (59.8985)	529 (53.4597)	
College graduate or above	788 (27.163)	472 (27.2464)	316 (30.0399)	
**BMI**				
<25	739 (25.474)	661 (40.6677)	78 (6.9187)	<0.0001
25–30	1,094 (37.7111)	726 (41.3712)	368 (31.9661)	
≥30	1,068 (36.8149)	361 (17.9611)	707 (61.1153)	
**Smoking consumption status**				
Former	934 (32.1958)	504 (28.8722)	430 (36.4388)	0.0021
Never	1,427 (49.1899)	898 (54.1694)	529 (46.8338)	
Now	540 (18.6143)	346 (16.9584)	194 (16.7273)	
**Alcohol consumption status**				
Never	321 (11.0651)	172 (8.4513)	149 (9.7817)	<0.0001
Former	609 (20.9928)	312 (14.5500)	297 (23.7675)	
Mild	1,088 (37.5043)	674 (44.7740)	414 (40.3806)	
Moderate	419 (14.4433)	295 (17.9888)	124 (11.5295)	
Heavy	464 (15.9945)	295 (14.2359)	169 (14.5407)	
**Physical activity**				
<600	601 (20.717)	324 (17.3104)	277 (22.1800)	0.0227
≥600	2,300 (79.283)	1,424 (82.6896)	876 (77.8200)	
**Life’s Essential 8**				
Low	229 (7.8938)	50 (1.9466)	179 (12.8678)	<0.0001
Moderate	2,115 (72.9059)	1,192 (63.4380)	923 (81.4893)	
High	557 (19.2003)	506 (34.6153)	51 (5.6430)	
**Health behaviors score**				
Low	308 (10.617)	171 (8.2436)	137 (12.0450)	<0.0001
Moderate	1,378 (47.5009)	783 (42.5378)	595 (51.9826)	
High	1,215 (41.8821)	794 (49.2186)	421(35.9724)	
**Health factors score**				
Low	569 (19.6139)	116 (4.7558)	453 (35.5187)	<0.0001
Moderate	1706 (58.8073)	1,045 (56.7313)	661 (60.1887)	
High	626 (21.5788)	587 (38.5129)	39 (4.2926)	
Life’s Essential 8	70.4533 (0.5150)	74.7462 (0.4409)	62.8636 (0.6173)	<0.0001
Health behaviors score	73.4767 (0.6039)	74.7458 (0.6573)	71.2329 (0.7711)	<0.001
Health factors score	67.4299 (0.6235)	74.7467 (0.5072)	54.4943 (0.7522)	<0.0001
Score HEI	44.6330 (1.3662)	47.1105 (1.4445)	40.2528 (1.7384)	<0.001
Score PA	93.2066 (0.3677)	93.7918 (0.4480)	92.1720 (0.6145)	0.0369
Score Smoke	72.5890 (1.1198)	73.5542 (1.3825)	70.8824 (1.3309)	0.1164
Score Sleep	83.4782 (0.6036)	84.5267 (0.6699)	81.6245 (0.9869)	0.0135
Score BMI	62.4746 (0.9148)	73.8701 (0.8935)	42.3281 (1.0948)	<0.0001
Score Non-HDL	56.8598 (0.7420)	61.3074 (0.9865)	48.9966 (1.0709)	<0.0001
Score Glucose	84.2873 (0.7510)	90.6622 (0.5858)	73.0170 (1.2865)	<0.0001
Score BP	66.0980 (1.0482)	73.1472 (1.1810)	53.6353 (1.4524)	<0.0001
Phenotypic age	49.7488 (0.3331)	47.4360 (0.3644)	53.8377 (0.5152)	<0.0001
Serum Klotho	844.1060 (9.2288)	850.9989 (11.4185)	831.9197 (11.7211)	0.1904
Biological age	53.7941 (0.3638)	51.7649 (0.4245)	57.3818 (0.4323)	<0.0001
SII	553.1432 (6.9480)	553.3166 (10.4497)	552.8367 (11.2500)	0.9777
DII	1.2480 (0.0695)	1.1409 (0.0798)	1.4373 (0.0864)	0.0037
Age, years	54.8763 (0.2980)	53.8139 (0.3448)	56.7545 (0.4235)	<0.0001

Non-MetS-ATP, Non-Metabolic syndrome-Adult Treatment Panel-III; MetS-ATP, Metabolic syndrome-Adult Treatment Panel-III; BMI, Body Mass Index; NHANES, National Health and Nutrition Examination Survey; FBG, fasting blood glucose; HEI, Healthy Eating Index; Non-HDL, Non-High-Density Lipoprotein Cholesterol; BP, Blood Pressure; SII, Systemic Immune-Inflammatory Index and DII, Dietary Inflammatory Index. Data are Mean (standard error) or No. of Participants (Weighted %). ^a^Percentages were adjusted for NHANES survey weights. The *P*-value was calculated using a chi-square test and Students T test after considering the sampling weights.

Table 1 summarizes all participant demographics. Among the 2,901 participants included in this study (48.2% female), a mean age of 54.9 years, and a mean LE8 score of 70.4533 (standard error of 0.5150). 1, 153 individuals were diagnosed with MetS. Participants with MetS were older, had a higher BMI, and were married compared to those without MetS. The MetS group had lower LE8 scores and had higher Phenotypic age, Biological age, and DII.

As depicted in Supplementary Figure 2, there were also strong differences in the MetS-ATP and related components among MetS and non-MetS subjects. In particular, MetS subjects exhibited substantially enhanced hyperglycemia, abnormal TG, abnormal HDL, and obesity, relative to non-MetS, however, the hypertensive incidences were similar between MetS and non-MetS participants.

### 3.2 Univariate logistic regression model

Based on our LE8 and related component univariate analyses, biological aging and inflammatory markers were strongly associated with MetS risk (Table 2). Apart from the PA score, smoke score, circulating Klotho and SII, other covariates showed marked association with MetS development (*p* < 0.05).

**Table 2:** Univariate logistic regression analysis.

Parameter	OR (95% CI)	*P*-value	*P*-trend
Life’s Essential 8	0.9069 (0.8941,0.9200)	<0.0001	<0.001
Health behaviors score	0.9867 (0.9789,0.9944)	0.0024	0.001
Health factors score	0.9082 (0.8978,0.9187)	<0.0001	<0.001
Score HEI	0.9925 (0.9885,0.9966)	0.0014	0.008
Score PA	0.9947 (0.9887,1.0008)	0.0821	0.369
Score Smoke	0.9985 (0.9957,1.0014)	0.2948	0.268
Score Sleep	0.9955 (0.9915,0.9996)	0.0324	0.004
Score BMI	0.9641 (0.9603,0.9680)	<0.0001	<0.001
Score Non-HDL	0.9852 (0.9812,0.9892)	<0.0001	<0.001
Score Glucose	0.9704 (0.9659,0.9750)	<0.0001	<0.001
Score BP	0.9798 (0.9757,0.9840)	<0.0001	<0.001
Phenotypic age	1.0779 (1.0502,1.1063)	<0.0001	<0.001
Serum Klotho	0.9998 (0.9994,1.0002)	0.3386	0.960
Biological age	1.0913 (1.0714,1.1116)	<0.0001	<0.001
SII	0.9999 (0.9995,1.0003)	0.5549	0.230
DII	1.0773 (1.0234,1.1341)	0.0075	<0.001
**Life’s Essential 8**			
Low	ref	ref	<0.001
Moderate	0.1751 (0.0986,0.3109)	<0.0001	
High	0.0223 (0.0106,0.0468)	<0.0001	
**Health behaviors score**			
Low	ref	ref	<0.001
Moderate	0.8150 (0.5514,1.2047)	0.2805	
High	0.4743 (0.3054,0.7367)	0.0027	
**Health factors score**			
Low	ref	ref	<0.001
Moderate	0.1403 (0.0897,0.2194)	<0.0001	
High	0.0148 (0.0077,0.0285)	<0.0001	

HEI, healthy eating index; BMI, Body Mass Index; PA, Physical activity; Non-HDL, non-high-density lipoprotein cholesterol; BP, blood pressure; SII, Systemic Immune-Inflammatory Index and DII, Dietary Inflammatory Index; Adjusted for Sex, Age, Ethnic/race, Marital, Family income-to-poverty ratio, Education levels and Alcohol consumption status.

### 3.3 Multivariate logistic regression model

**Figure 1:**
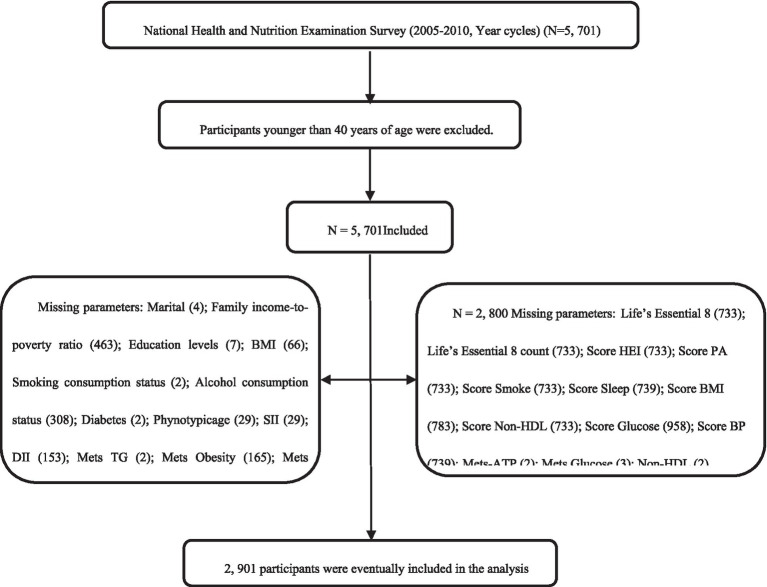
Flow chart of the screening process for the selection of the study population. Non-MetS-ATP, Non-Metabolic syndrome-Adult Treatment Panel-III; MetS-ATP, Metabolic syndrome-Adult Treatment Panel-III; BMI, Body Mass Index; NHANES, National Health and Nutrition Examination Survey; FBG, fasting blood glucose; HEI score, Healthy Eating Index; Non-HDL, Non-High-Density Lipoprotein Cholesterol; BP, Blood Pressure; SII, Systemic Immune-Inflammatory Index and DII, Dietary Inflammatory Index.

Table 3 depicts the conclusions of our multivariate analysis of LE8 and its components with MetS. Following possible confounding factor adjustment, we developed several models for evaluation of the association between LE8 and MetS. We revealed that, with a rise in the LE8 and health factor scores, there was a drastic reduction in MetS risk (*P-*trend < 0.05). The MetS incidence odds ratios (95% confidence intervals) among LE8 middle and high scoring cohorts were (OR = 0.5163, 95Cl 0.2818~0.9460) and (OR = 0.2027, 95Cl 0.0871~0.4714), respectively, relative to the LE8 low scoring cohort. Furthermore, relative to the low health factor scores, the MetS odds ratios (95% confidence interval) among middle and high health factor scores were (OR = 0.1737, 95%Cl 0.1080~0.2794), (OR = 0.0278, 95%Cl 0.0130~0.0591) respectively (Figure 1). Using the 8 LE8 components as continuous variables, and following confounding factors adjustment, we revealed that 4 health factor scores were inversely proportional to MetS risk, and the OR values were as follows: BMI (OR = 0.9663, 95%Cl 0.9598~0.9728), Glucose (OR = 0.9768, 95%Cl 0.9678~0.9859), BP (OR = 0.9813, 95%Cl 0.9724~0.9903), and Non-HDL (OR = 0.9843, 95%Cl 0.9768~0.9918).

**Table 3:** Multiple logistic regression models of Life’s Essential 8 with MetS-ATP for participants.

Parameter	Crude model		Model 1		Model 2	
	OR (95%CI)	*P*-value	OR (95%CI)	*P*-value	OR (95%CI)	*P*-value
**Life’s Essential 8**						
Low	ref		ref		ref	
Moderate	0.5230 (0.3131,0.8736)	0.0153	0.5061 (0.2920,0.8771)	0.0176	0.5163(0.2818,0.9460)	0.0353
High	0.2063 (0.0964,0.4416)	<0.001	0.1955 (0.0903,0.4232)	<0.001	0.2027(0.0871,0.4714)	0.0018
P-trend	<0.001		<0.001		<0.001	
**Health behaviors score**						
Low	ref		ref		ref	
Moderate	0.9865 (0.7032,1.3839)	0.9349	0.9650(0.6805,1.3683)	0.8339	0.9744 (0.6390,1.4857)	0.8936
High	0.8834 (0.5865,1.3305)	0.5392	0.7791(0.5085,1.1936)	0.2371	0.8052 (0.4837,1.3402)	0.3656
P-trend	<0.001		<0.001		<0.001	
**Health factors score**						
Low	ref		ref		ref	
Moderate	0.1791 (0.1187,0.2703)	<0.0001	0.1740 (0.1134,0.2669)	<0.0001	0.1737 (0.1080,0.2794)	<0.0001
High	0.0287 (0.0146,0.0562)	<0.0001	0.0282 (0.0140,0.0567)	<0.0001	0.0278 (0.0130,0.0591)	<0.0001
P-trend	<0.001		<0.001		<0.001	
**Life’s Essential 8**						
Score HEI	0.9991 (0.9942,1.0040)	0.6958	0.9983 (0.9932,1.0035)	0.4915	0.9984 (0.9906,1.0062)	0.5510
Score PA	0.9982 (0.9912,1.0052)	0.5891	0.9993 (0.9916,1.0071)	0.8569	0.9994 (0.9874,1.0115)	0.8787
Score Smoke	0.9955 (0.9926,0.9984)	0.0039	0.9952 (0.9920,0.9984)	0.0058	0.9951 (0.9897,1.0006)	0.065
Score Sleep	1.0027 (0.9988,1.0067)	0.1683	1.0005 (0.9960,1.0049)	0.8248	1.0004 (0.9939,1.0069)	0.8628
Score BMI	0.9673 (0.9631,0.9716)	<0.0001	0.9665 (0.9621,0.9709)	<0.0001	0.9663 (0.9598,0.9728)	<0.001
Score Non-HDL	0.9846 (0.9799,0.9893)	<0.0001	0.9848 (0.9799,0.9896)	<0.0001	0.9843 (0.9768,0.9918)	0.0069
Score Glucose	0.9780 (0.9730,0.9831)	<0.0001	0.9768 (0.9709,0.9828)	<0.0001	0.9768 (0.9678,0.9859)	0.004
Score BP	0.9821 (0.9762,0.9879)	<0.0001	0.9810 (0.9747,0.9873)	<0.0001	0.9813 (0.9724,0.9903)	0.0072
Phenotypic age	1.0145 (0.9898,1.0397)	0.2366	1.0221(0.9879,1.0574)	0.1900	1.0204 (0.9715,1.0717)	0.2824
Serum Klotho	0.9999 (0.9995,1.0002)	0.5016	0.9999 (0.9995,1.0003)	0.5588	0.9998 (0.9993,1.0004)	0.4420
Biological age	1.0021 (0.9756,1.0292)	0.8742	1.0049 (0.9797,1.0307)	0.6876	1.0045 (0.9668,1.0437)	0.7324
SII	0.9996 (0.9993,1.0000)	0.0547	0.9994 (0.9990,0.9999)	0.0121	0.9994 (0.9988,1.0000)	0.0587
DII	1.0271 (0.9576,1.1015)	0.4341	1.0118 (0.9391,1.0902)	0.7405	1.0070 (0.8970,1.1305)	0.8601

HEI, healthy eating index; BMI, Body Mass Index; PA, Physical activity; Non-HDL, non-high-density lipoprotein cholesterol; BP, blood pressure; SII, Systemic Immune-Inflammatory Index and DII, Dietary Inflammatory Index; Crude model, No adjustment for any potential influence factors. Model 1, Adjusted for Sex, Age and Ethnic/race. Model 2, Adjusted for Sex, Age, Ethnic/race, Marital, Family income-to-poverty ratio Education levels and Alcohol consumption status.

Scoring the LE8 scores and health factors and health behaviors as low, medium and high, after adjusting for the covariates of Sex, Age and Ethnic/race in Model 1, the ORs of participants in the high LE8 grouping and the high health factors grouping compared to the low scoring group were (OR = 0.1955, 95% Cl 0.0903,0.4232), (OR = 0.0282, 95%Cl 0.0140,0.0567) (*p* < 0.05 for trend), and after further adjusting for Marital, Family income-to-poverty ratio Education levels and Alcohol consumption status, the associations in model 2 remained unchanged, with a 79.73% reduction in MetS risk for the high subgroup of LE8 compared to the low subgroup, OR = 0.2027 (95%Cl 0.0871,0.4714), and a 97.22% reduction in MetS risk for the high subgroup of health factors compared to the low subgroup, OR = 0.0278 (95%Cl 0.0130,0.0591). When the eight components of LE8 were used as continuous variables, it was observed that after adjusting for confounders, the four health factor scores were negatively associated with MetS, with OR values of Score BMI (OR = 0.9663, 95% Cl 0.9598~0.9728), Score Glucose (OR = 0.9768, 95% Cl 0.9678~0.9859), Score BP (OR = 0.9813, 95%Cl 0.9724~0.9903), and Score Non-HDL (OR = 0.9843, 95%Cl 0.9768~0.9918), whereas Health Behavior as a whole and its four components were not significantly associated with MetS (*p* > 0.05).

**Figure 2:**
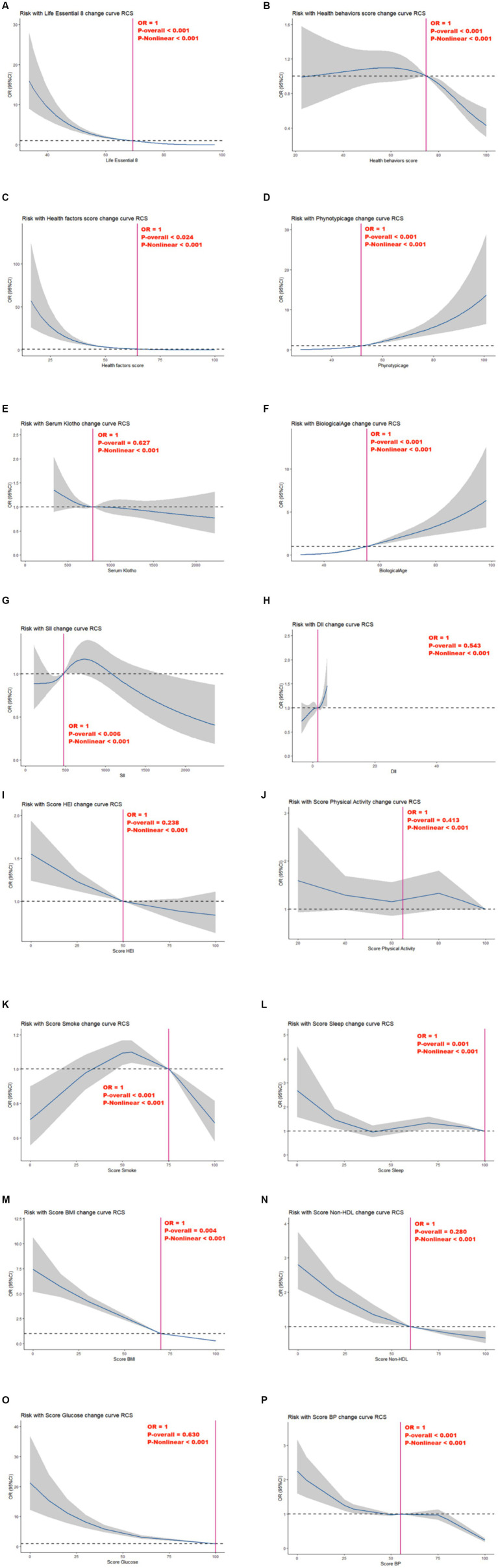
Relationships between Life’s Essential 8 scores **(A)**, health behavior score **(B)**, health factor score **(C)**, Phenotypic age **(D)**, Serum Klotho **(E)**, Biological Age **(F)**, SII **(G)**, DII **(H)**, Score HEI **(I)**, Score Physical Activity **(J)**, Score Smoke **(K)**, Score Sleep **(L)**, Score BMI **(M)**, Score Non-HDL **(N)**, Score Glucose **(O)**, Score BP **(P)** and MetS. OR (95% CI; shaded areas) were adjusted for Sex, Age, Ethnic/race, Marital, Family income-to-poverty ratio Education levels and Alcohol consumption status. Vertical red solid lines indicate the minimal threshold for the beneficial association with estimated OR = 1. OR, odds ratio.

As presented in Figure 2, following confounding factor adjustment, a nonlinear association was discovered between MetS risk and several variables, including, the LE8 scores, health behavior scores, health factor scores, phenotype age, circulating Klotho, biological age, SII, DII, HEI, Physical Activity, Smoke, Sleep, BMI, Non-HDL, Glucose, and BP (*P*-nonlinear <0.001; estimated OR = 1).

Nonetheless, we observed no marked independent correlation between circulating Klotho, DII, HEI, physical activity, non-HDL, and glucose and MetS risk in the overall model (*P*-overall > 0.05).

### 3.4 Subgroup analysis involving LE8 and MetS

Table 4 summarizes the subgroup analysis results from LE8 and MetS risk. Suggested that the risk of MetS prevalence significantly decreased with progressively higher LE8 and health factor group scores across gender, race, household economic status, BMI, physical activity, and alcohol intake (*P*-trend < 0.05). In case of women, whites, other races, household income-to-poverty ratio ≥ 5, BMI ≥ 30, and heavy drinkers, elevation in health behavior scores drastically decreased MetS risk (*p* < 0.05 for trend).

**Table 4:** Results of multiple logistic regression of participant scores Life’s Essential 8 with MetS-ATP subgroup analysis.

Parameter	Life’s Essential 8					
	Low	Moderate		High		*P*-trend
	ref	OR (95%CI)	*P*-value	OR (95%CI)	*P*-value	
**Sex**						
Male	ref	0.1298 (0.0578,0.2918)	<0.0001	0.0204(0.0064,0.0650)	<0.0001	<0.0001
Female	ref	0.2334 (0.1093,0.4987)	<0.001	0.0235 (0.0099,0.0554)	<0.0001	<0.0001
**Ethnicity/Race**						
White	ref	0.1954 (0.0880,0.4337)	<0.001	0.0258 (0.0102,0.0654)	<0.0001	<0.0001
Black	ref	0.1744 (0.0754,0.4037)	<0.001	0.0298 (0.0070,0.1272)	<0.001	<0.001
Mexican	ref	0.2536 (0.0505,1.2729)	0.0886	0.0162 (0.0039,0.0679)	<0.0001	<0.0001
Other	ref	0.0441 (0.0104,0.1860)	<0.001	0.0018 (0.0002,0.0136)	<0.0001	<0.0001
**Ratio of family income to poverty levels**						
<1.3	ref	0.2536 (0.1507,0.4268)	<0.0001	0.0129 (0.0041,0.0409)	<0.0001	<0.0001
1.3–3	ref	0.1264 (0.0510,0.3131)	<0.001	0.0123 (0.0035,0.0431)	<0.0001	<0.0001
3–5	ref	0.1626 (0.0480,0.5505)	0.0061	0.0098 (0.0020,0.0473)	<0.0001	<0.0001
≥5	ref	0.1612 (0.0436,0.5954)	0.0089	0.0303 (0.0071,0.1300)	<0.0001	<0.0001
**BMI**						
<25	ref	0.3517 (0.0855,1.4465)	0.1361	0.0716 (0.0126,0.4050)	0.0055	0.0049
25–30	ref	0.3320 (0.0928,1.1879)	0.0851	0.0815 (0.0174,0.3806)	0.0034	<0.001
≥30	ref	0.2292 (0.1192,0.4406)	<0.001	0.0466 (0.0168,0.1293)	<0.0001	<0.0001
**Physical activity**						
<600	ref	0.2241 (0.0944,0.5324)	0.0022	0.0000 (0.0000,0.0000)	<0.0001	<0.0001
≥600	ref	0.1574 (0.0719,0.3447)	<0.001	0.0220 (0.0085,0.0572)	<0.0001	<0.0001
**Alcohol consumption status**						
Heavy	ref	0.1681 (0.0718,0.3936)	<0.001	0.0060 (0.0010,0.0356)	<0.0001	<0.0001
Mild	ref	0.1727 (0.0586,0.5085)	0.0030	0.0182 (0.0050,0.0656)	<0.0001	<0.0001
Never	ref	0.2078 (0.0323,1.3359)	0.0927	0.0129 (0.0012,0.1457)	0.0015	<0.001
Former	ref	0.2023 (0.0863,0.4740)	<0.001	0.0403 (0.0097,0.1669)	<0.001	<0.001
Moderate	ref	0.1155 (0.0293,0.4557)	0.0038	0.0296 (0.0068,0.1298)	<0.0001	<0.001

Adjusted for sex, age, ethnic/race, marital, family income-to-poverty ratio education levels and alcohol consumption status.

Furthermore, we also assessed associations between MetS and 2 inflammatory indices and 3 biological aging indicators. Based on our analysis, biological and phenotypic ages were strongly associated with MetS risk in various gender, race, family economic status, BMI, physical activity and alcohol intake populations (*p* < 0.05; Supplementary Tables 3A,B). Higher levels of CVH were associated with lower risk of MetS compared to low CVH (*P*-trend<0.05), with the strongest association being physical activity, with a high CVH OR = 0.0000 (95%Cl 0.0000,0.0000) compared to low CVH in the <600 min/month group. Further analysis of its components (health factors and health behaviors) in LE8, see Supplementary Tables 2A,B, similarly saw the same trend of negative associations between health factor scores and MetS across subgroups (*P-*trend < 0.05). In contrast, the negative association of health behavior scores with MetS was correlated among participants who were female, white, had a household income to poverty ratio of ≥5, BMI ≥ 30, Physical Activity ≥ 600, and heavy drinkers, with ORs for high-scoring health behaviors compared to low-scoring health behaviors of (0.4676, 95%Cl 0.2669,0.8191), (0.5399, 95%Cl 0.3285,0.8873), (0.3444,95%Cl 0.1251,0.9483), (0.3790, 95%Cl 0.2073,0.6928), (0.5829, 95%Cl 0.3279,1.0362), (0.3206, 95%Cl 0.1728, 0.5948) (*P-trend* < 0.05).

The association of two inflammatory indices and three biological aging indicators with MetS was also examined in subgroup analyses, suggesting that biological age and phenotypic age were positively associated with MetS among participants in different gender, race, household economic status, BMI, physical activity, and alcohol intake groups (*p* < 0.05; Supplementary Tables 3A,B).

### 3.5 Mediation analyses

We employed parallel mediation assessment to assess modulatory roles of biological aging (phenotypic age, circulating Klotho, biological age) and inflammatory index (SII, DII) in the intricate relationship between the LE8, health behavior, and health factor scores and MetS risk.

Of note, phenotypic age exhibited a strong modulatory role on the relationship between LE8, health behavior, and health factor scores and MetS risk, with mediation proportions 0.13%, *p* ≤ 0.001; 0.0.69%, *p* = 0.240; 0.08%; and *p* ≤ 0.001, respectively.

Biological age exhibited a strong modulatory role on the relationship between LE8 and MetS risk, with mediation proportions at 0.08%, *p* ≤ 0.001 (Figure 2).

### 3.6 Sensitivity analysis

**Figure 3:**
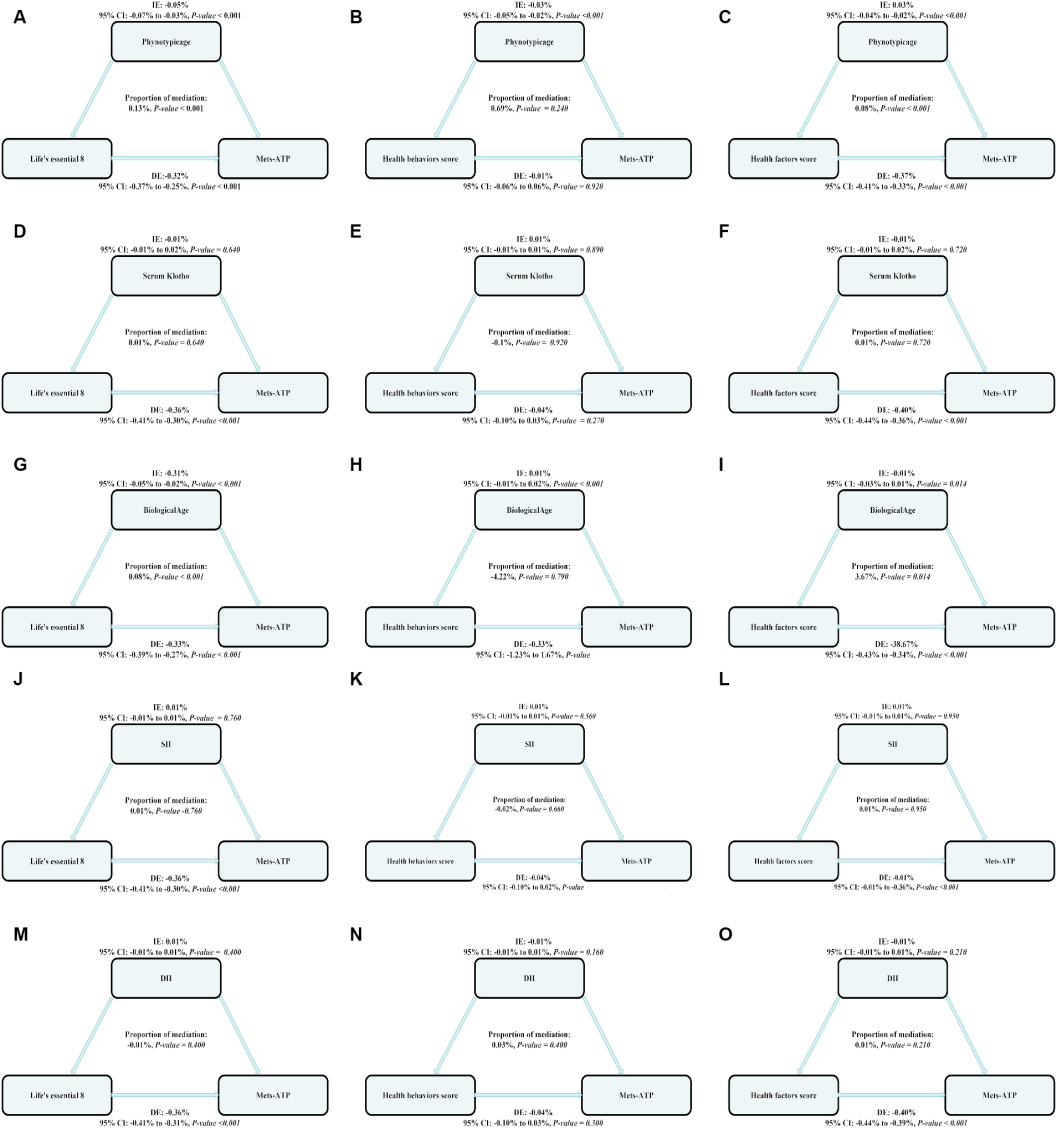
The estimated proportions of the associations between LE8, health behavior score, health factor score and MetS mediated effect by the Biological aging (Phenotypic age, Serum Klotho, Biological age) and inflammation index (SII, DII). Model adjusted for Sex, Age, Ethnic/race, Marital, Family income-to-poverty ratio Education levels and Alcohol consumption status. IE, the estimate of the indirect effect; DE, the estimate of the direct effect; Proportion of mediation = IE/DE + IE, OR, odds ratio. Panels **(A–C)** show the relationship between LE8, health behavior, and health factor scores and MetS: the mediating effect of Phenotypic age. Panels **(D–F)** show the relationship between LE8, health behavior, and health factor scores and MetS: the mediating effect of Serum Klotho. Panels **(G–I)** show the relationship between LE8, health behavior, and health factor scores and MetS: the mediating effect of Biological age. Panels **(J–L)** show the relationship between LE8, health behavior, and health factor scores and MetS: the mediating effect of SII. Panels **(M–O)** show the relationship between LE8, health behavior, and health factor scores and MetS: the mediating effect of DII.

A new MetS definition was presented at the International Diabetes Federation (IDF) workshop in 2005, which was distinct from the ATP III description in that it emphasized central obesity as a prerequisite (23). In 2009, IDF renewed the MetS definition yet again, reducing the importance of waist measurement from 2006. Under the new definition, waist size was only used as a preliminary screening tool, with strict thresholds set for the waist circumference, based on distinct ethnic populations (24). Given that certain articles employed the IDF-based MetS diagnosis in 2005 and 2009, sensitivity analysis was conducted to validate whether distinct diagnostic criteria impacted the conclusions of this investigation (Supplementary Tables 4–7). We revealed robust inverse relationships between LE8 and health factors and MetS even under different diagnostic criteria (Figure 3).

## 4 Discussion

In the current study, we provide two main novel findings based on the US general population; first, increasing LE8 scores in middle-aged and older adults (≥40 years old) reduces the risk of developing MetS. In addition, two aging indicators, Phenotypic age and Biological age, were identified as mediators of the positive association between LE8 and MetS risk.

Herein, we employed the National Cholesterol Education Program (NCEP)-based ATPIII criteria to diagnose MetS. In short, a participant was diagnosed with MetS if ≥3 out of the following 5 metabolic abnormalities were present: central obesity, increased TG content, diminished HDL-cholesterol contents, increased BP, and high fasting glucose levels (25). The aforementioned factors are also robust hazard factors for cardiovascular disease, Based on previous studies, indicating a strong link between MetS and cardiovascular disease risk (26). With rising economic development and a rapidly increasing elderly population, the MetS incidence is also rising, which poses a substantial health challenge to individuals and the public. MetS has multiple risk factors. At present, it is linked with lifestyle (27), aging (28) and low-grade inflammation (29). Among the aforementioned risk factors, unhealthy lifestyle namely, unhealthy diet (increased high-fat and high-sugar food intake), sedentary, and lack of exercise are modifiable factors that contribute to various lipid metabolic disorders, insulin resistance and liver steatosis, which, in turn, accelerates MetS development (25). LE8 is a quantifiable cardiovascular health (CVH) evaluation index, and it is otherwise known as “healthy eating, more activity, smoking cessation, adequate sleep, weight control, cholesterol management, BP control and diabetes hypoglycemia management,” in prior investigations, however, an LE8 and MetS correlation was hardly examined. The conclusions from this investigation are follows: a rise in LE8 scores among middle-aged and older adults is highly protective against MetS. Moreover, enhanced LE8 and health factor scores drastically decreased MetS risk. However, the relationship between LE8 and MetS has rarely been mentioned in previous studies. Our study provides the first epidemiologic evidence that LE8 scores are negatively associated with MetS among middle-aged and older adults. A recent study provided similar findings in a recent investigation (30), it was revealed that improvements in the 4 health factors in LE8 can profoundly diminish hypertension and diabetes risks among the general population, BP and blood sugar control can highly benefit (i.e., reduce) MetS risk. An IDF consensus (31) states that MetS is easily managed by encouraging healthy lifestyle choices, such as, healthy eating and proper increases in physical activity, which, in turn, enhance LE8 scores. Hence, MetS risk can potentially be diminished by enhancing LE8 scores, which, in turn, CVH.

In case of MetS mechanism, we focused on inflammatory. MetS is typically marked with reduced-grade chronic inflammation in all energy homeostasis-related tissues, such as, adipose tissue, islets and liver (32). Hotamisligil et al. (33) reported that an inflammatory cytokine TNF-α is highly expressed in the fat tissues of obese animal models. This was the first report that linked inflammation to obesity. In subsequent investigations, it was revealed that TNF-α is also upregulated in the fat and muscle tissues of obese individuals (34). SII is a newly discovered inflammatory biomarker that integrates 3 principal blood cell lineages and accurately represents inflammation complexity (15). Considering the association between inflammation and metabolic diseases, SII is gaining much interest in predicting metabolic diseases like cardiovascular diseases. An analysis of the NHANES database (35) suggested a strong correlation between augmented SII (abdominal obesity, hypertension and HDL-C) and MetS risk. In another study, diet was reported to be key in developing chronic inflammation (36). DII is a highly dependable biomarker that measures the impact of dietary factors on individual inflammatory response. Several investigations demonstrated an intricate link between DII and MetS, however, there is controversy in the results. One survey from the Korean KNHANES database (37) revealed that DII is strongly related to MetS frequency in men and postmenopausal women. However, an emerging study reported no marked association between inflammatory potential of DII-evaluated diets and MetS risk. Herein, we demonstrated a strong direct association between augmented DII scores and MetS risk among middle-aged and older adults, thereby indicating that decreasing DII scores (using diet adjustments) can potentially prevent the onset and progression of MetS among middle-aged and older populations.

Based on the aforementioned statistical results, we next performed mediation analysis for the two inflammatory biomarkers. We revealed that both SII and DII did not modulate the relationship between MetS and 4 health behaviors and 4 health factors in LE8. This indicated that LE8 may modulate MetS using other channels.

Aging is a possible mechanism linked with MetS. Aging occurs as a result of several multifaceted and complicated factors that cannot solely be explained by the physiological decline over time. In fact, this alteration is strongly associated with several age-associated human chronic diseases, and not the actual age of the individual. Distinct from the real age, certain Biological aging indicators, namely, Biological age, Phenotypic age and circulating Klotho as reliable aging indicators, as reported in multiple clinical studies (38–40). However, no prior investigations associated the aforementioned 3 aging bioindicators with MetS risk. Herein, We first linked phenotypic age, Serum Klotho, and biological age to MetS among middle-aged and elderly individuals. Using single factor analysis, it was revealed that both phenotypic and biological ages were intricately linked to MetS. Following confounding factors adjustment, namely, patient sex, age, ethnicity/race, marital status, family income to poverty ratio, education status, alcohol consumption status, phenotypic age and biological age were still directly associated with MetS risk among the middle-aged and elderly population in various subgroups. In addition, we observed no marked correlation between circulating Klotho and MetS risk among middle-aged and elderly patients. We further performed mediation analyses for the phenotypic age, circulating Klotho levels, and biological age. We revealed that the phenotypic age mediated strong associations between LE8, health behavior, and health factor scores and MetS risk, with proportions 0.13%, *p* ≤ 0.001;0.0.69%, *p* = 0.240;0.08%; and *p* ≤ 0.001, respectively. The biological age also mediated the relationship between LE8 scores and MetS risk, with 0.08% ratio, *p* ≤ 0.001. A study involving the NHANES US population (41) reported a marked inverse association between LE8 score and phenotypic age progression rate. In particular, they observed a substantial decrease in phenotypic age of 1.14 years for every 10-point rise in the LE8 score. These results were strongly corroborated in another investigation, indicating that the LE8, health behavior and health factor scores were inversely proportional to the phenotypic age, biological age, with evidences of stronger correlations between health factors and MetS risk, and the correlation was stronger for health factors, which is similar to our findings (42). Improving health factors and healthy behaviors, such as, good BMI, lipids, blood sugar and BP can significantly enhance the LE8 scores, and assist in slowing down the aging process. According to emerging reports (43), maintaining a proper BMI can significantly enhance physical function among older adults, reduce metabolic diseases, and related medical costs, while increasing healthy life expectancy. Another investigation (44) revealed that human lipids are critical modulators of longevity, and maintaining ideal lipid status is a robust anti-aging strategy. Additionally, in a cross-sectional investigation involving 288 adults aged ≥ 50 years (45), it was reported that hypertension is strongly related to a rapidly increased epigenetic age, and this followed a dose-dependent pattern, confirming that BP management is an efficacious anti-aging approach. The correlation between hyperglycemia and aging has been validated in multiple studies: a 2-year prospective cohort investigation (46) revealed that MetS, prediabetic, and diabetic patients were at an enhanced cognitive impairment risk. In conclusion, the above evidence supports our finding that improved LE8 may regulate aging. In addition, studies have pointed out that MetS is profiled as a series of distinct metabolic abnormalities related to oxidative stress and significantly diminished antioxidant protection mechanisms. These conditions reduce telomere lengths, Telomere length is exacerbated by the cumulative effects of its components, such as obesity, high blood sugar and high blood pressure, and in presence of extremely short telomere lengths in ≥1 chromosomes, the genomic integrity is lost, which increases cell proliferation restriction via processes like senescence and apoptosis (47, 48). This, in turn, worsens MetS progression. Improving LE8 health behaviors, for example, jogging, increasing consumption of fiber diet, and adequate sleep, can significantly increase endogenous antioxidant expressions, which may delay telomere attrition, reduces oxidative stress and mitochondrial dysfunction (49–51), thereby delaying MetS progression. According to the above, our findings provide a better understanding of the role of aging in the relationship between LE8 and MetS.

As the understanding of MetS deepens, the diagnostic criteria for MetS are constantly being updated. The MetS definition was renewed at the IDF workshop in 2006, by emphasizing central obesity as a prerequisite to MetS (5). In 2009, IDF again revised the MetS definition, reducing the relevance of waist measurement. The waist size was only used as a preliminary screening tool, with discreet waist circumference cut-offs for people of different ethnic origins (52). To verify whether the different diagnostic criteria affected the results of this study, we performed a sensitivity analysis, We found a significant inverse association between LE8 and health factors and risk of MetS whether using the diagnostic criteria for 2006 or 2009 IDF.

Among the strengths of this investigation is that this was the first to analyze the link between augmented LE8 scores and MetS in a relatively large patient population. Secondly, we demonstrated that 2 inflammatory and 3 aging markers strongly modulated the relationship between LE8 scores and MetS risk. Nonetheless, this research has certain limitations. Firstly, this was a cross-sectional investigation involving United States adult residents over 40 years of age. Thus, it is nearly impossible to establish a precise chronological order or eliminate cases involving reverse causality and internal bias. Secondly, the NHANES trial has multiple internal biases. Thirdly, it is likely that there were residual and unconsidered confounding factors and test errors that may have affected the outcomes of this study. Fourthly, despite adjusting the survey cycle, the duration of our analysis was insufficient and may have introduced bias in our results. Lastly, we conducted mediation analysis using a cross-sectional study design, therefore, it was challenging to infer causality. Considering the aforementioned limitations, our conclusions must be perceived with caution, and additional investigation is necessary to further support our findings.

## 5 Conclusion

This study showed a negative correlation between LE8 score and MetS among middle-aged and elderly individual, and the negative association of both is partly mediated by biological aging.

A rise in the LE8 score s protects against MetS. In fact, elevated LE8 and health factor scores strongly diminish MetS risk. Furthermore, biological aging-associated markers, such as, Phenotypic age, Biological age and MetS incidence were directly correlated among middle-aged and elderly individuals. Moreover, using mediation analyses, we revealed that the LE8 and MetS risk association was potentially modulated by biological aging. Thus, we identified aging as a risk factor for MetS, and highlighted anti-aging approaches as a potential way to increase LE8 and protect against MetS incidence.

## Data availability statement

The datasets presented in this study can be found in online repositories. The names of the repository/repositories and accession number(s) can be found in the article/Supplementary material.

## Ethics statement

The studies involving humans were approved by the NCHS Research Ethics Review Board. The studies were conducted in accordance with the local legislation and institutional requirements. The participants provided their written informed consent to participate in this study.

## Author contributions

RG: Conceptualization, Data curation, Visualization, Writing – review & editing. SX: Resources, Software, Supervision, Writing – original draft. XL: Validation, Writing – original draft. HW: Visualization, Writing – original draft. SQ: Data curation, Investigation, Writing – original draft. BL: Formal analysis, Writing – original draft. CL: Conceptualization, Funding acquisition, Project administration, Writing – review & editing. JC: Methodology, Project administration, Resources, Visualization, Writing – review & editing.
