# Supplementary material for: Relationship between Life’s Essential 8 and metabolic syndrome among older Americans (NHANES, 2007–2010): navigating biological aging and inflammation
Source: Front Med (Lausanne). 2024 Jun 5;11:1380464. doi: 10.3389/fmed.2024.1380464 (PMC11188479; doi:10.3389/fmed.2024.1380464)
Supplement: Supplementary file 1 [file Data_Sheet_1.PDF]

Supplementary Table 1. Definition and scoring approach for the American Heart Association's Life's Essential 8 score.

Domain	CVH Metric	Measurement	Quantification and Scoring of CVH Metric
Health Behaviors	Diet	Healthy Eating Index-2015 diet score percentile	Quantiles of DASH-style diet adherence<br><br>**Scoring (Population):**<br><u>Points</u> <u>Quantile</u><br>100 ≥95th percentile (top/ideal diet)<br>80 75th – 94th percentile<br>50 50th – 74th percentile<br>25 25th – 49th percentile<br>0 1st – 24th percentile (bottom/least ideal quartile)
	Physical activity	Self-reported minutes of moderate or vigorous physical activity per week	**Metric:** Minutes of moderate (or greater) intensity activity per week<br><br>**Scoring:**<br><u>Points</u> <u>Minutes</u><br>100 ≥150<br>90 120 – 149<br>80 90 – 119<br>60 60 – 89<br>40 30 – 59<br>20 1 – 29<br>0 0
	Nicotine exposure	Self-reported use of cigarettes or inhaled nicotine- delivery system	**Metric:** Combustible tobacco use and/or inhaled NDS use; or secondhand smoke exposure<br><br>**Scoring:**<br><u>Points</u> <u>Status</u>

			100 Never smoker<br>75 Former smoker, quit ≥5 yrs<br>50 Former smoker, quit 1 - <5 yrs<br>25 Former smoker, quit <1 year, or currently using inhaled NDS<br>0 Current smoker<br><br>Subtract 20 points (unless score is 0) for living with active indoor smoker in home
	Sleep health	Self-reported average hours of sleep per night	**Metric:** Average hours of sleep per night<br><br>**Scoring:**<br><u>Points Level</u><br>100 7 – <9<br>90 9 – <10<br>70 6 – <7<br>40 5 – <6 or ≥10<br>20 4 – <5<br>0 <4
Health Factors	Body mass index	Body weight (kg) divided by height squared (m^2)	**Metric:** Body mass index (kg/m^2)<br><br>**Scoring:** <u>Points Level</u><br>100 <25<br>70 25.0 – 29.9<br>30 30.0 – 34.9<br>15 35.0 – 39.9<br>0 ≥40.0

	Blood lipids	Plasma total and HDL-cholesterol with calculation of non-HDL-cholesterol	**Metric:** Non-HDL-cholesterol (mg/dL)<br><br>**Scoring:**<br><u>Points Level</u><br>100 <130<br>60 130 – 159<br>40 160 – 189<br>20 190 – 219<br>0 ≥220<br><br>If drug-treated level, subtract 20 points
	Blood glucose	Fasting blood glucose or casual hemoglobin A1c	**Metric:** Fasting blood glucose (mg/dL) or Hemoglobin A1c (%)<br><br>**Scoring:**<br><u>Points Level</u><br>100 No history of diabetes and FBG <100 (or HbA1c < 5.7)<br>60 No diabetes and FBG 100 – 125 (or HbA1c 5.7-6.4) (Pre-diabetes)<br>40 Diabetes with HbA1c <7.0<br>30 Diabetes with HbA1c 7.0 – 7.9<br>20 Diabetes with HbA1c 8.0 – 8.9<br>10 Diabetes with Hb A1c 9.0 – 9.9<br>0 Diabetes with HbA1c ≥10.0
	Blood pressure	Appropriately measured systolic and diastolic blood pressure	**Metric:** Systolic and diastolic blood pressure (mm Hg)<br><br>**Scoring:**<br><u>Points Level</u><br>100 <120/<80 (Optimal)

			75 120-129/<80 (Elevated)<br>50 130-139 or 80-89 (Stage I HTN)<br>25 140-159 or 90-99<br>0 ≥160 or ≥100<br><br>Subtract 20 points if treated level

Supplementary Table 2a Results of multiple logistic regression of participant Scores Health Behaviors with Mets-ATP subgroup analysis.

Parameter	Health Behaviors Score					
	Low	Moderate		High		*P-trend*
	ref	OR (95%CI)	*P-value*	OR (95%CI)	*P-value*	
**Sex**						
Male	ref	0.8687 (0.5204,1.4502)	0.5686	0.6368 (0.3840,1.0560)	0.0768	0.0258
Female	ref	0.8626 (0.5432,1.3700)	0.508	0.4676 (0.2669,0.8191)	0.011	0.002
**Ethnicity/ Race**						
white	ref	0.9248 (0.5749,1.4876)	0.7329	0.5399 (0.3285,0.8873)	0.018	<0.001
black	ref	1.0294 (0.5617,1.8868)	0.9171	0.9081 (0.3976,2.0739)	0.8	0.7434
Mexican	ref	1.5372 (0.5401,4.3752)	0.388	1.1852 (0.4047,3.4704)	0.7364	0.5951
other	ref	0.2402 (0.1091,0.5286)	0.0017	0.2988 (0.0989,0.9029)	0.0344	0.1465
**Ratio of family income to poverty levels**						
<1.3	ref	1.0962 (0.5611,2.1414)	0.7759	0.8009 (0.4114,1.5592)	0.4915	0.4939
1.3-3	ref	1.0161 (0.5327,1.9382)	0.9591	0.7211 (0.3481,1.4935)	0.3579	0.2028
3-5	ref	0.8693 (0.4819,1.5680)	0.6215	0.4867 (0.2234,1.0604)	0.0676	0.022
≥5	ref	0.5476 (0.2160,1.3884)	0.1907	0.3444 (0.1251,0.9483)	0.0402	0.0066
**BMI**						
**<25**	ref	0.6154 (0.2045,1.8518)	0.3624	0.6670 (0.2451,1.8154)	0.4023	0.4981

**25-30**	ref	0.7203 (0.4141,1.2529)	0.2257	0.4017 (0.2033,0.7939)	0.0121	0.0039
**>=30**	ref	0.4555 (0.2599,0.7983)	0.0092	0.3790 (0.2073,0.6928)	0.0037	0.0103
**Physical Activityg**						
**<600**	ref	0.9706 (0.5693,1.6549)	0.9068	0.5107 (0.2081,1.2535)	0.1315	0.116
**≥600**	ref	0.8566 (0.4795,1.5303)	0.5780	0.5829 (0.3279,1.0362)	0.0640	0.0052
**Alcohol consumption status**						
heavy	ref	0.4098 (0.2215,0.7582)	0.0068	0.3206 (0.1728,0.5948)	0.0011	0.0017
mild	ref	1.3023 (0.7512,2.2578)	0.3277	0.7644 (0.3966,1.4735)	0.4023	0.0201
never	ref	1.3409 (0.2694,6.6737)	0.7046	0.6340 (0.1186,3.3896)	0.5738	0.1042
former	ref	1.2253 (0.4852,3.0938)	0.6514	0.7279 (0.2435,2.1759)	0.5511	0.296
moderate	ref	0.8153 (0.2627,2.5307)	0.7101	0.6770(0.1990,2.3026)	0.5128	0.4348

Abbreviations: Mets-ATP, Metabolic syndrome- Adult Treatment Panel-III; BMI, Body Mass Index.

Supplementary Table 2b Results of multiple logistic regression of participant Scores Health Factors with Mets-ATP subgroup analysis.

	**Health Factor Score**					
**Parameter**	**Low**	**Moderate**		**High**		***P-trend***
	**ref**	**OR (95%CI)**	***P-value***	**OR (95%CI)**	***P-value***	
**Sex**						
Male	ref	0.1339 (0.0795,0.2257)	<0.0001	0.0109 (0.0057,0.0211)	<0.0001	<0.0001

Female	ref	0.1366 (0.0757,0.2466)	<0.0001	0.0146 (0.0064,0.0335)	<0.0001	<0.0001
**Ethnicity/ Race**						
white	ref	0.1408 (0.0845,0.2347)	<0.0001	0.0157 (0.0073,0.0339)	<0.0001	<0.0001
black	ref	0.1933 (0.1010,0.3702)	<0.001	0.0071 (0.0008,0.0639)	<0.001	<0.0001
Mexican	ref	0.1426 (0.0700,0.2906)	<0.0001	0.0084 (0.0018,0.0386)	<0.0001	<0.0001
other	ref	0.0400 (0.0151,0.1062)	<0.0001	0.0005 (0.0000,0.0087)	<0.0001	<0.0001
**Ratio of family income to poverty levels**						
<1.3	ref	0.1670 (0.0852,0.3272)	<0.0001	0.0123 (0.0059,0.0255)	<0.0001	<0.0001
1.3-3	ref	0.0752 (0.0401,0.1411)	<0.0001	0.0097 (0.0029,0.0321)	<0.0001	<0.0001
3-5	ref	0.1607 (0.0709,0.3642)	<0.001	0.0223 (0.0077,0.0646)	<0.0001	<0.0001
≥5	ref	0.1778 (0.0849,0.3723)	<0.001	0.0140 (0.0047,0.0417)	<0.0001	<0.0001
**BMI**						
**<25**	ref	0.0730 (0.0091,0.5848)	0.0171	0.0087 (0.0009,0.0863)	<0.001	<0.0001
**25-30**	ref	0.1771 (0.0967,0.3242)	<0.0001	0.0427 (0.0160,0.1136)	<0.0001	<0.0001
**>=30**	ref	0.2607 (0.1523,0.4462)	<0.0001	0.0260 (0.0059,0.1150)	<0.001	<0.0001
**Physical Activityg**						
**<600**	ref	0.1368 (0.0670,0.2795)	<0.0001	0.0080 (0.0021,0.0302)	<0.0001	<0.0001
**≥600**	ref	0.1369 (0.0851,0.2204)	<0.0001	0.0155 (0.0076,0.0314)	<0.0001	<0.0001
**Alcohol consumption status**						
heavy	ref	0.1055 (0.0350,0.3181)	<0.001	0.0150 (0.0035,0.0651)	<0.0001	<0.0001
mild	ref	0.1025 (0.0530,0.1984)	<0.0001	0.0094 (0.0035,0.0252)	<0.0001	<0.0001
never	ref	0.1417 (0.0416,0.4826)	0.0037	0.0018 (0.0002,0.0183)	<0.0001	<0.0001
former	ref	0.1988 (0.1123,0.3516)	<0.0001	0.0237 (0.0077,0.0736)	<0.0001	<0.0001
moderate	ref	0.1765 (0.0840,0.3705)	<0.001	0.0207 (0.0049,0.0876)	<0.0001	<0.0001

Abbreviations:Mets-ATP, Metabolic syndrome- Adult Treatment Panel-III; BMI, Body Mass Index.

Supplementary Table 3a Results of multiple logistic regression of participant Biological aging with Mets-ATP subgroup analysis.

Parameter	Biological aging					
	Phynotypicage		Serum Klotho		BiologicalAge	
	OR (95%CI)	*P-value*	OR (95%CI)	*P-value*	OR (95%CI)	*P-value*
**Sex**						
Male	1.0393 (1.0236,1.0553)	<0.0001	1.0001 (0.9994,1.0008)	0.7595	1.0460 (1.0304,1.0619)	<0.0001
Female	1.0492 (1.0373,1.0613)	<0.0001	0.9995 (0.9990,0.9999)	0.0237	1.0472 (1.0347,1.0598)	<0.0001
**Ethnicity/ Race**						
white	1.0414 (1.0291,1.0538)	<0.0001	0.9998 (0.9993,1.0003)	0.4257	1.0453(1.0345,1.0562)	<0.0001
black	1.0510 (1.0299,1.0724)	<0.001	0.9997 (0.9990,1.0003)	0.2679	1.0449 (1.0186,1.0719)	0.0030
Mexican	1.0518 (1.0340,1.0699)	<0.0001	0.9996 (0.9985,1.0006)	0.3977	1.0681 (1.0460,1.0907)	<0.0001
other	1.0594 (1.0274,1.0923)	0.0011	0.9994 (0.9981,1.0007)	0.3221	1.0538 (1.0197,1.0889)	0.0039
**Ratio of family income to poverty levels**						
<1.3	1.0389 (1.0254,1.0525)	<0.0001	0.9995 (0.9990,1.0000)	0.0375	1.0364 (1.0221,1.0509)	<0.0001
1.3-3	1.0429 (1.0267,1.0594)	<0.0001	0.9993 (0.9985,1.0002)	0.1038	1.0406 (1.0233,1.0582)	<0.0001
3-5	1.0483 (1.0309,1.0659)	<0.0001	0.9993 (0.9984,1.0001)	0.0770	1.0505 (1.0303,1.0711)	<0.0001
≥5	1.0431 (1.0239,1.0628)	<0.001	1.0006 (1.0000,1.0012)	0.0514	1.0546 (1.0328,1.0768)	<0.0001
**Alcohol consumption status**						
heavy	1.0420 (1.0214,1.0631)	<0.001	0.9998 (0.9991,1.0006)	0.6328	1.0642 (1.0422,1.0867)	<0.0001
mild	1.0378 (1.0247,1.0510)	<0.0001	0.9998 (0.9991,1.0004)	0.5084	1.0418 (1.0272,1.0567)	<0.0001
never	1.0523 (1.0195,1.0860)	0.0033	0.9995 (0.9985,1.0005)	0.3205	1.0576 (1.0334,1.0822)	<0.0001
former	1.0494 (1.0261,1.0732)	<0.001	0.9997 (0.9989,1.0005)	0.4257	1.0426 (1.0176,1.0682)	0.0019
moderate	1.0590 (1.0362,1.0822)	<0.0001	0.9997 (0.9988,1.0007)	0.5584	1.0600 (1.0344,1.0863)	<0.0001

Abbreviations:Mets-ATP, Metabolic syndrome- Adult Treatment Panel-III.

Supplementary Table 3b Results of multiple logistic regression of participant Biological aging with Mets-ATP subgroup analysis.

Parameter	inflammation index			
	SII		DII	
	OR (95%CI)	*P-value*	OR (95%CI)	*P-value*
**Sex**				
Male	0.9995 (0.9990,1.0000)	0.0528	1.0629 (0.9825,1.1499)	0.1203
Female	1.0002 (0.9997,1.0007)	0.4116	1.0778 (0.9959,1.1664)	0.0618
**Ethnicity/ Race**				
white	0.9998 (0.9994,1.0003)	0.4578	1.0751 (1.0174,1.1361)	0.0129
black	1.0001 (0.9991,1.0011)	0.8095	0.9636 (0.8434,1.1009)	0.5530
Mexican	0.9998 (0.9993,1.0003)	0.3051	0.9401 (0.8418,1.0499)	0.2486
other	1.0000(0.9992,1.0008)	0.9938	1.2032 (1.0066,1.4383)	0.0431

**Ratio of family income to poverty levels**				
<1.3	1.0003 (0.9999,1.0007)	0.1849	1.0563 (0.9443,1.1815)	0.3180
1.3-3	0.9994 (0.9988,1.0000)	0.0412	1.0230 (0.9385,1.1152)	0.5873
3-5	1.0001 (0.9994,1.0008)	0.7371	1.0772 (0.9365,1.2389)	0.2778
≥5	0.9997 (0.9989,1.0005)	0.3979	1.0967 (0.9634,1.2484)	0.1523
**Alcohol consumption status**				
heavy	0.9996 (0.9984,1.0008)	0.4844	1.0624 (0.8853,1.2749)	0.4964
mild	1.0002 (0.9996,1.0008)	0.5025	1.0464 (0.9555,1.1460)	0.3106
never	1.0001 (0.9991,1.0010)	0.8470	1.1343 (0.9475,1.3580)	0.1585
former	0.9996 (0.9991,1.0001)	0.1386	1.1027 (0.8920,1.3630)	0.3477
moderate	0.9997 (0.9987,1.0008)	0.5986	1.0888 (0.9360,1.2666)	0.2544

Abbreviations:Mets-ATP, Metabolic syndrome- Adult Treatment Panel-III; SII, Systemic Immune-Inflammatory Index and DII, Dietary Inflammatory Index.

Supplementary Table 4 Participant demographic characteristics (NHANES, 2007- 2010 Year Cycle).

parameter	No. of Participants (Weighted %) [a]			*P-value*
	**Total (N= 1, 447)**	**Non-Mets-IDF2006 (N= 666)**	**Mets-IDF2006 (N=781)**	
Sex				
Female	706 (48.7906)	346 (55.3694)	360(45.9254)	0.0181
Male	741 (51.2094)	320 (44.6306)	421(54.0746)	
Ethnicity/ Race				
White people	841 (58.1202)	393 (82.3097)	448(80.3457)	0.5396
Black people	221 (15.273)	105 (7.3967)	116(7.1802)	
Mexican	205 (14.1672)	86 (4.0647)	119(4.9394)	
other	180 (12.4395)	82 (6.2289)	98(7.5347)	
Marital				
Married	969 (66.9661)	458 (74.0450)	511(72.2226)	0.7667
Separated	367 (25.3628)	155 (19.5741)	212(20.6528)	
Never married	111 (7.671)	53 (6.3809)	58(7.1246)	
Ratio of family income to poverty levels				
<1.3	371 (25.6393)	149 (11.7775)	222(16.3331)	0.0112
1.3-3	412 (28.4727)	183 (22.1767)	229(24.7318)	
3-5	324 (22.3912)	151 (25.4049)	173(27.7332)	
≥5	340 (23.4969)	183 (40.6409)	157(31.2019)	
Education levels				
Less than 11th grade	341 (23.566)	138 (11.7163)	203(16.4539)	0.0087
High school graduate	720 (49.7581)	357 (62.7632)	363(53.7348)	
College graduate or above	386 (26.6759)	171 (25.5205)	215(29.8113)	
BMI				
<25	370 (25.5701)	290 (45.5733)	80(8.9881)	<0.0001
25-30	557 (38.4934)	250 (37.5513)	307(41.0592)	

>=30	520(35.9364)	126 (16.8754)	394(49.9527)	
Smoking consumption status				
Former	483 (33.3794)	195 (28.9216)	288(38.3431)	
Never	701 (48.4451)	348 (54.4933)	353(46.0568)	0.0201
Now	263 (18.1755)	123 (16.5850)	140(15.6001)	
Alcohol consumption status				
Never	309 (21.3545)	126 (14.6664)	183(20.3263)	
Former	228 (15.7567)	115 (16.0407)	113(12.5127)	
Mild	559 (38.6317)	261 (43.4750)	298(44.5936)	0.0927
Moderate	209 (14.4437)	107 (18.3028)	102(14.1594)	
Heavy	142 (9.8134)	57 (7.5151)	85(8.4079)	
Physical Activityg				
<600	306 (21.1472)	132 (19.3230)	174(21.4924)	0.4793
≥600	1141 (78.8528)	534 (80.6770)	607(78.5076)	
Life's essential 8				
Low	115 (7.9475)	14 (1.5698)	101 (9.9539)	
Moderate	1034 (71.4582)	408 (55.3011)	626 (82.1663)	<0.0001
High	298 (20.5943)	244 (43.1290)	54 (7.8799)	
Health behaviors score				
Low	156 (10.7809)	56 (6.7641)	100 (11.3457)	
Moderate	688 (47.5466)	296 (41.2591)	392 (52.0864)	0.0023
High	603 (41.6724)	314 (51.9768)	289 (36.5679)	
Health factors score				
Low	253 (17.4845)	17 (1.5530)	236 (25.1244)	
Moderate	852 (58.8804)	364 (51.2679)	488 (65.8025)	<0.0001
High	342 (23.6351)	285 (47.1791)	57 (9.0731)	
Life's essential 8	71.0059 (0.5357)	76.8940 (0.6467)	65.0896 (0.5461)	<0.0001
Health behaviors score	73.2645 (0.7320)	75.3269 (1.0187)	71.1922 (0.7234)	<0.001
Health factors score	68.7473 (0.6626)	78.4610 (0.7653)	58.9870 (0.6621)	<0.0001

Score HEI	44.1840 (1.4055)	47.9248 (1.8948)	40.4253 (1.8234)	0.0054
Score PA	92.3973 (0.6658)	92.8211 (0.8896)	91.9715 (0.7603)	0.3952
Score Smoke	72.6250 (1.4245)	74.1740 (2.1671)	71.0686 (1.7025)	0.2571
Score Sleep	83.8516 (0.8064)	86.3878 (1.0986)	81.3033 (1.0252)	0.0013
Score BMI	62.4113 (1.1728)	75.9189 (1.7547)	48.8390 (1.1357)	<0.0001
Score Non-HDL	57.5681 (0.9206)	62.1736 (1.2453)	52.9405 (0.9956)	<0.0001
Score Glucose	86.3226 (0.7586)	95.3273 (0.8173)	77.2747 (1.1094)	<0.0001
Score BP	68.6871 (1.2916)	80.4243 (1.6320)	56.8936 (1.3714)	<0.0001
Phenotypic age	49.3429 (0.3615)	45.5010 (0.4966)	53.2033 (0.4120)	<0.0001
Serum Klotho	844.2388 (12.4729)	845.1865 (18.9529)	843.2866 (14.7952)	0.935
Biological age	53.2323 (0.4471)	49.4344 (0.5105)	57.0485 (0.4688)	<0.0001
SII	539.7887 (10.5638)	533.4478 (20.0801)	546.1599 (11.7568)	0.618
DII	1.2466 (0.0872)	1.0476 (0.1228)	1.4466 (0.0915)	0.0049
Age, years	54.6620 (0.3690)	52.4794 (0.4464)	56.8550 (0.4136)	<0.0001

Abbreviations: Non-Mets-IDF2006, Non-Metabolic syndrome- International Diabetes Federation2005; Mets-IDF2005, Metabolic syndrome- International Diabetes Federation2005; BMI, Body Mass Index; NHANES, National Health and Nutrition Examination Survey; FBG, fasting blood glucose; HEI score, Healthy Eating Index; Non-HDL, Non-High-Density Lipoprotein Cholesterol; BP, Blood Pressure; SII, Systemic Immune-Inflammatory Index and DII, Dietary Inflammatory Index.

Data are Mean (standard error) or No. of Participants (Weighted %).

[a] *Percentages were adjusted for NHANES survey weights. The P-value was calculated using a chi-square test and Students T test after considering the sampling weights.*

Supplementary Table 5 Multiple logistic regression models of Life's Essential 8 with Mets-IDF2005 for participants.

Parameter	Crude model		Model 1		Model 2	
	OR (95%CI)	*P-value*	OR (95%CI)	*P-value*	OR (95%CI)	*P-value*
**Life's Essential 8**						
**Low**	**ref**		**ref**		**ref**	
**Moderate**	0.7927 (0.3241,1.9385)	0.5978	0.6886 (0.2704,1.7539)	0.416	0.6738 (0.2439,1.8614)	0.4069
**High**	0.2179 (0.0843,0.5627)	0.0028	0.1812 (0.0676,0.4859)	0.0017	0.1751 (0.0597,0.5139)	0.0048
P-trend	<0.001		<0.001		<0.001	
**Health Behaviors Score**						
**Low**	**ref**		**ref**		**ref**	
**Moderate**	0.7719 (0.4516,1.3197)	0.3302	0.6984 (0.4026,1.2113)	0.1896	0.6987 (0.3725,1.3107)	0.2329
**High**	0.8081 (0.5224,1.2499)	0.3244	0.6716 (0.4161,1.0840)	0.0985	0.6595 (0.3736,1.1639)	0.1336
P-trend	<0.001		<0.001		<0.001	
**Health Factors Score**						
**Low**	**ref**		**ref**		**ref**	
**Moderate**	0.0981 (0.0423,0.2277)	<0.0001	0.0987 (0.0423,0.2306)	<0.0001	0.0976 (0.0380,0.2509)	<0.001
**High**	0.0246 (0.0103,0.0589)	<0.0001	0.0292 (0.0121,0.0705)	<0.0001	0.0287 (0.0108,0.0763)	<0.0001
P-trend	<0.001		<0.001		<0.001	

Crude model, No adjustment for any potential influence factors.

Model 1, Adjusted for Sex, Age and Ethnic/race.

Model 2, Adjusted for Sex, Age, Ethnic/race, Marital, Family income-to-poverty ratio Education levels and Alcohol consumption status.

Supplementary Table 6 Participant demographic characteristics (NHANES, 2007- 2010 Year Cycle).

parameter	No. of Participants (Weighted %) [a]			*P-value*
	Total (N= 2, 901)	Non-Mets-IDF2009 (N= 1, 563)	Mets-IDF2009 (N=1, 338)	
Sex				

Female	1397 (48.1558)	785 (52.6325)	612 (46.5548)	0.0176
Male	1504 (51.8442)	778 (47.3675)	726 (53.4452)	
Ethnicity/ Race				
White people	1672 (57.6353)	925 (81.3922)	747 (80.3813)	0.1251
Black people	447 (15.4085)	255 (7.5271)	192 (7.0108)	
Mexican	426 (14.6846)	191 (4.0857)	235 (5.8638)	
other	356 (12.2716)	192 (6.9950)	164 (6.7441)	
Marital				
Married	1964 (67.7008)	1070 (73.7855)	894 (73.1988)	0.4125
Separated	728 (25.0948)	364 (19.4497)	364 (21.0565)	
Never married	209 (7.2044)	129 (6.7649)	80 (5.7447)	
Ratio of family income to poverty levels				
<1.3	706 (24.3364)	338 (11.4651)	368 (16.0649)	<0.001
1.3-3	868 (29.9207)	447 (22.4101)	421 (25.9745)	
3-5	620 (21.3719)	351 (25.5843)	269 (25.6094)	
≥5	707 (24.3709)	427 (40.5405)	280 (32.3512)	
Education levels				
Less than 11th grade	695 (23.9573)	332 (12.7695)	363 (16.1823)	0.0032
High school graduate	1418 (48.8797)	811(60.4149)	607 (53.4983)	
College graduate or above	788 (27.163)	420 (26.8156)	368 (30.3193)	
BMI				
<25	739 (25.474)	605 (41.1367)	134 (10.3286)	<0.0001
25-30	1094 (37.7111)	613 (40.0480)	481 (35.0000)	
>=30	1068 (36.8149)	345 (18.8153)	723 (54.6714)	
Smoking consumption status				
Former	934 (32.1958)	452 (28.9678)	482 (35.3867)	0.0104
Never	1427 (49.1899)	804 (54.1375)	623 (47.7668)	

Now	540 (18.6143)	307 (16.8948)	233 (16.8465)	
Alcohol consumption status				
Never	609 (20.9928)	273(14.5889)	336 (22.5968)	
Former	464 (15.9945)	269(14.5850)	195 (14.0035)	
Mild	1088 (37.5043)	610(44.8533)	478 (40.7984)	<0.0001
Moderate	419 (14.4433)	255(17.5735)	164 (12.9059)	
Heavy	321 (11.0651)	156(8.3993)	165 (9.6954)	
Physical Activityg				
<600	601 (20.717)	292(17.2873)	309 (21.6241)	0.0304
≥600	2300 (79.283)	1271(82.7127)	1029 (78.3759)	
Life's essential 8				
Low	229 (7.8938)	43 (1.9069)	186 (11.6037)	
Moderate	2115 (72.9059)	1039 (61.9142)	1076 (81.4897)	<0.0001
High	557 (19.2003)	481 (36.1789)	76 (6.9066)	
Health behaviors score				
Low	308 (10.617)	145 (7.8016)	163 (12.2187)	
Moderate	1378 (47.5009)	706 (42.8825)	672 (50.3462)	<0.0001
High	1215 (41.8821)	712 (49.3159)	503 (37.4352)	
Health factors score				
Low	569 (19.6139)	95 (4.4580)	474 (32.2245)	
Moderate	1706 (58.8073)	910 (55.2623)	796 (61.8759)	<0.0001
High	626 (21.5788)	558 (40.2798)	68 (5.8996)	
Life's essential 8	70.4533 (0.5150)	75.1222 (0.4734)	63.7621 (0.6161)	<0.0001
Health behaviors score	73.4767 (0.6039)	74.8388 (0.6572)	71.5245 (0.8040)	<0.001
Health factors score	67.4299 (0.6235)	75.4056 (0.5254)	55.9996 (0.7048)	<0.0001
Score HEI	44.6330 (1.3662)	47.172 3 (1.4411)	40.9937 (1.7071)	<0.001
Score PA	93.2066 (0.3677)	93.9318 (0.3934)	92.1673 (0.6393)	0.0183
Score Smoke	72.5890 (1.1198)	73.6286 (1.4226)	71.0991 (1.3185)	0.1397
Score Sleep	83.4782 (0.6036)	84.6226 (0.7311)	81.8380 (0.9507)	0.0233

Score BMI	62.4746 (0.9148)	73.5852 (0.9352)	46.5515 (1.1195)	<0.0001
Score Non-HDL	56.8598 (0.7420)	62.1473 (1.0390)	49.2819 (0.9307)	<0.0001
Score Glucose	84.2873 (0.7510)	91.6557 (0.5163)	73.7273 (1.2456)	<0.0001
Score BP	66.0980 (1.0482)	74.2341 (1.1919)	54.4377 (1.2121)	<0.0001
Phenotypic age	49.7488 (0.3331)	47.0995 (0.3643)	53.5457 (0.5226)	<0.0001
Serum Klotho	844.1060 (9.2288)	850.8209 (11.4164)	834.4825 (10.8658)	0.2087
Biological age	53.7941 (0.3638)	51.3486 (0.4077)	57.2989 (0.4366)	<0.0001
SII	553.1432 (6.9480)	554.9389 (10.3088)	550.5697 (10.6200)	0.7845
DII	1.2480 (0.0695)	1.1435 (0.0848)	1.3977 (0.0780)	0.0086
Age, years	54.8763 (0.2980)	53.5429 (0.3463)	56.7872 (0.4153)	<0.0001

Abbreviations: Non-Mets-IDF2009, Non-Metabolic syndrome- International Diabetes Federation2009; Mets-IDF2009, Metabolic syndrome- International Diabetes Federation2009; BMI, Body Mass Index; NHANES, National Health and Nutrition Examination Survey; FBG, fasting blood glucose; HEI score, Healthy Eating Index; Non-HDL, Non-High-Density Lipoprotein Cholesterol; BP, Blood Pressure; SII, Systemic Immune-Inflammatory Index and DII, Dietary Inflammatory Index.

Data are Mean (standard error) or No. of Participants (Weighted %).

a Percentages were adjusted for NHANES survey weights. The P-value was calculated using a chi-square test and Students T test after considering the sampling weights.

Supplementary Table 7 Multiple logistic regression models of Life's Essential 8 with Mets-IDF2009 for participants.

Parameter	Crude model		Model 1		Model 2	
	**OR (95%CI)**	***P-value***	**OR (95%CI)**	***P-value***	**OR (95%CI)**	***P-value***
**Life's Essential 8**						
**Low**	**ref**		**ref**		**ref**	
**Moderate**	0.6205 (0.3722,1.0344)	0.066	0.6041 (0.3517,1.0374)	0.0661	0.6116 (0.3328,1.1238)	0.1019
**High**	0.2448 (0.1213,0.4940)	<0.001	0.2378 (0.1178,0.4799)	<0.001	0.2457 (0.1125,0.5367)	0.0025
P-trend	<0.001		<0.001		<0.001	
**Health Behaviors Score**						
**Low**	**ref**		**ref**		**ref**	
**Moderate**	0.8160 (0.5619,1.1849)	0.2726	0.7709 (0.5227,1.1370)	0.1783	0.7672 (0.4875,1.2074)	0.2221
**High**	0.8153 (0.5245,1.2673)	0.3501	0.6981 (0.4424,1.1015)	0.1161	0.7005 (0.4079,1.2028)	0.1730
P-trend	<0.001		<0.001		<0.001	

**Health Factors Score**						
**Low**	**ref**		**ref**		**ref**	
**Moderate**	0.1891 (0.1233,0.2901)	<0.0001	0.1870 (0.1202,0.2911)	<0.0001	0.1875 (0.1155,0.3045)	<0.0001
**High**	0.0376 (0.0225,0.0629)	<0.0001	0.0397 (0.0234,0.0671)	<0.0001	0.0393 (0.0221,0.0699)	<0.0001
P-trend	<0.001		<0.001		<0.001	

Crude model, No adjustment for any potential influence factors.

Model 1, Adjusted for Sex, Age and Ethnic/race.

Model 2, Adjusted for Sex, Age, Ethnic/race, Marital, Family income-to-poverty ratio Education levels and Alcohol consumption status.
